# Cancer risk in road transportation workers: a national representative cohort study with 600,000 person-years of follow-up

**DOI:** 10.1038/s41598-020-68242-5

**Published:** 2020-07-09

**Authors:** Wanhyung Lee, Mo-Yeol Kang, Jihyun Kim, Sung-Shil Lim, Jin-Ha Yoon

**Affiliations:** 1 Department of Occupational and Environmental Medicine, Gil Medical Center, Gachon University College of Medicine, Incheon, Republic of Korea; 2 Department of Occupational and Environmental Medicine, Seoul St. Mary’s Hospital, College of Medicine, The Catholic University of Korea, Seoul, Republic of Korea; 3 Graduate School of Public Health, Yonsei University, Seoul, Republic of Korea; 4 The Institute for Occupational Health, Department of Preventive Medicine, Yonsei University College of Medicine, 50, Yonsei-ro, Seodaemun-gu, Seoul, 03722 Republic of Korea; 5 Department of Preventive Medicine, Yonsei University College of Medicine, Seoul, Republic of Korea

**Keywords:** Cancer prevention, Risk factors

## Abstract

We analysed cancer risk in road transportation workers (RTWs) exposed to traffic air pollution and motor vehicle engine exhaust using the Korean National Health Insurance Service database. RTWs were defined as individuals in the transportation workers group doing road transportation. First admission history of cancer within a 3-year wash-out period was defined as an incident case. The crude incidence, standardised incidence ratio (SIR), and 95% confidence interval (CI) of all cancer risk of RTWs were compared with those of government employees or the whole working population. In total, 3,074 cancer cases were found among RTWs. The respective SIRs and 95% CIs for cancers in RTWs compared with those in the whole population were as follows: liver and intrahepatic bile duct cancers, 1.15 and 1.04–1.27; other digestive organ cancers, 1.28 and 1.04–1.57; trachea, bronchus, and lung cancers, 1.28 and 1.15–1.43; and bladder cancer, 1.26 and 1.03–1.52, respectively. The corresponding SIRs and 95% CIs were also higher in RTWs than in government employees. RTWs have a high risk of developing cancer, including cancer in the liver, intrahepatic bile ducts, other digestive organs, trachea, bronchus, lung, and bladder. Our results can assist in establishing prevention strategies for various cancers in RTWs.

## Introduction

Road transport workers (RTWs) such as truck, bus, and taxi drivers account for approximately 3% of the working-age population in Korea^1^. RTWs face accident risks and injury by motor vehicles and are exposed to various chemicals, including motor engine exhaust^2^.

Motor engine exhaust is a complex mixture of particulates and gases^2^. Various hydrocarbons and derivatives (such as benzene, formaldehyde, toluene, and sulphur dioxide); inorganic sulphates and nitrates; metals, including lead and platinum; and polycyclic aromatic hydrocarbons (PAHs) make up the mixture of particulates and gases from vehicle engine exhaust^3^. The International Agency for Research on Cancer (IARC) reviewed the literature and categorised diesel engine exhaust (DEE) as a Group 1 carcinogen, indicating ‘carcinogenic to humans’, in 2012. They confirmed its carcinogenicity for lung cancer and reported a positive association with bladder cancer.

The IARC 2012 report showed that engine exhaust increased risk of cancers, such as brain tumours^4^, colorectal cancer^5^, and breast cancer^6^. Furthermore, animal studies and cellular experiments have shown that a significant relationship exists between engine exhaust and various cancer risks other than lung cancer^7^. One recent cohort study that assessed traffic air pollution exposure for 20 years showed an increased odds of lung, bladder, kidney, and prostate cancer^8^. Studies have shown when inhaled, fine particles can go through the systemic circulation and consequently cause DNA damage, which is linked to carcinogenesis^9–11^. However, there is a lack of studies about the association between traffic exhaust and gastrointestinal tract cancers. A well designed pooling analysis of four cohorts showed that the hazard ratio of liver cancer was elevated, but it did not reach statistical significance. Hence, comprehensive studies including all cancer risks related to RTWs are needed. Previous Asian studies with large sample sizes have demonstrated occupational classification-specific cancer risks^12–14^. However, those reports were based on data from the late 1990s or early 2000s and did not include various exposure scenarios. Thus far, research on focused cancer incidence in RTWs is limited.

Most well designed exposure assessments are based on a job-exposure matrix (JEM) such as NOCCA-JEMs^15^. JEM is not well-established in non-Western settings, and workers often change their jobs during working periods. Therefore, alterative study designs are needed. Therefore, with current dataset, we aimed to analyse all cancer risks faced by RTWs who are exposed to traffic engine exhaust. We used a national representative cohort study with around 600,000 person-years of RTW data to gain scientific evidence about cancer risks among RTWs. Our subgroup analysis that examines lifestyle differences can give more insight regarding epidemiological evidence. Furthermore, we undertook a sensitivity analysis by accounting for job change over the study period to determine bias of occupational exposure.

## Results

As shown in Table 1, from 2006 to 2015, there were 594,629 person-years for RTWs, 3,268,312 for government employees, and 62,760,615 for the whole working population. The most common age groups were 50–54 among RTWs (20.3%), 55–59 among government employees (19.0%), and 40–44 in the whole working population (18.3%).

**Table 1:** Characteristics of the study participants in the first year of the study (2006).

	Road transport workers		Reference group			
			Government employee		Whole working population	
Total person year	594,629		3,268,312		62,760,615	
Age (person year)						
25– < 30	11,039	(1.9)	57,160	(0.1)	4,240,331	(1.3)
30– < 35	34,743	(5.8)	210,875	(1.7)	9,019,025	(6.8)
35– < 40	66,560	(11.2)	390,711	(6.5)	11,470,377	(14.4)
40– < 45	94,738	(15.9)	516,571	(12.0)	10,897,150	(18.3)
45– < 50	117,612	(19.8)	615,957	(15.8)	9,434,555	(17.4)
50– < 55	120,496	(20.3)	622,025	(18.8)	7,639,526	(15.0)
55– < 60	91,293	(15.4)	484,914	(19.0)	5,170,518	(12.2)
60– < 65	46,769	(7.9)	279,612	(14.8)	3,111,404	(8.2)
65– < 70	11,379	(1.9)	90,487	(8.6)	1,777,729	(5.0)

Table 2 shows all cases of cancer, the crude incidence of all cancers per 100,000 persons, and the direct age-standardised incidence ratio (SIR) of all cancers and 95% confidence intervals [CIs] among RTWs during the study period. A total of 3,074 cases of cancer were found among RTWs. The annual crude incidence of all cancers per 100,000 persons was as low as 343.66 (2006) and as high as 481.15 (2013). The direct age-SIRs of all cancers were significantly higher in RTWs than in the whole working population in the study period.

**Table 2:** Age-standardised incidence ratios of all cancers—a comparison between the general working population and all road transportation workers in Korea by year.

Year	Cases of all cancer (n)	Road transport workers (n)	Crude incidence of all cancer per 100,000 persons	Age-standardised incidence ratio (95% CI)	
2006	207	60,234	343.66	2.40	(2.03–2.76)
2007	235	65,440	359.11	2.45	(2.09–2.82)
2008	276	68,560	402.57	2.80	(2.41–3.20)
2009	298	70,676	421.64	2.77	(2.40–3.14)
2010	342	73,375	466.10	2.73	(2.40–3.06)
2011	344	74,684	460.61	2.62	(2.29–2.95)
2012	336	75,103	447.39	2.60	(2.25–2.95)
2013	362	75,237	481.15	2.78	(2.40–3.15)
2014	320	75,084	426.19	2.13	(1.84–2.42)
2015	354	74,221	476.95	2.54	(2.19–2.89)

Age-standardised incidence ratio was calculated with direct standardisation methods for 5-year age groups.

**Figure 1:**
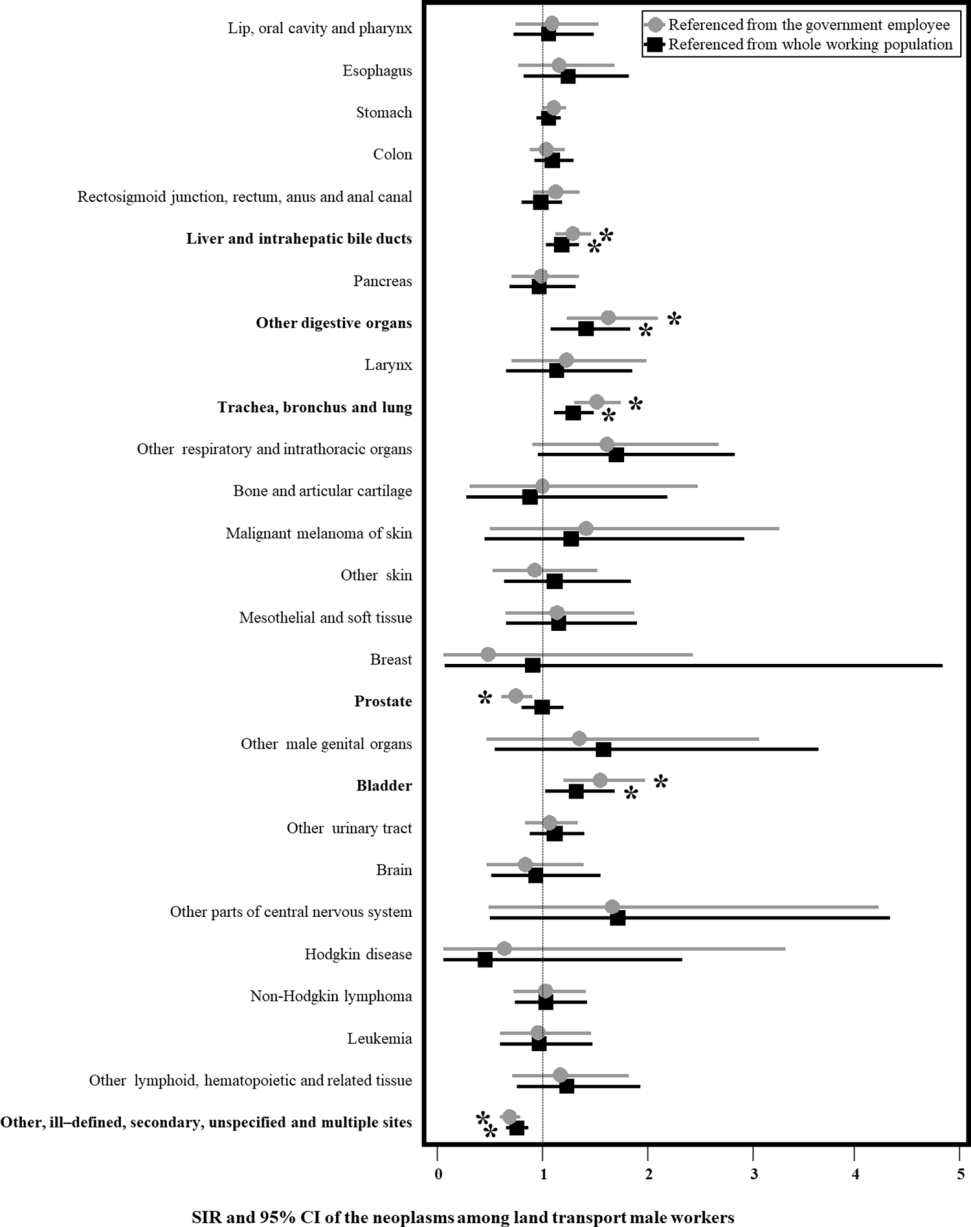
Standardised incidence ratio with 95% confidence intervals among land transport male workers reference from government employee and whole working population. This figure was drawn by author of WHL.

Each cancer risk with SIR and 95% CI in the first 1 year fixed exposure cohort is shown in Fig. 1. The cancer incidence was significantly higher in RTWs than in the referenced whole working population for cancers of the liver and intrahepatic bile ducts (SIR 1.15, 95% CI 1.04–1.27), other digestive organs (SIR 1.28, 95% CI 1.04–1.57), trachea, bronchus, and lung (SIR 1.28, 95% CI 1.15–1.43), and bladder (SIR 1.26, 95% CI 1.03–1.52). They were also higher when using government employees as a reference.

These significances were not attenuated when using the first 3 years fixed exposure and dynamic cohort definitions (Table 3), except for cancer of the liver and intrahepatic bile ducts referenced from the whole working population (SIR: 1.14, 95% CI 0.99–1.30).

**Table 3:** Age-standardised incidence ratio (SIR) and 95% confidence interval (CI) of cancers according to the different road transportation workers (RTWs).

	Definition of RTWs, SIR (95% CI)		
	First 1 year fixed RTWs	First 3 years fixed RTWs	More than 1 year worked as RTWs
**Referenced from the government employee**			
Liver and intrahepatic bile ducts neoplasms	1.23 (1.12–1.36)	1.24 (1.08–1.42)	2.41 (2.23–2.59)
Other digestive organs neoplasms	1.46 (1.18–1.78)	1.58 (1.19–2.06)	2.63 (2.25–3.06)
Trachea, bronchus and lung neoplasms	1.48 (1.33–1.65)	1.47 (1.26–1.70)	2.79 (2.58–3.02)
Bladder neoplasms	1.41 (1.16–1.71)	1.50 (1.15–1.93)	2.55 (2.20–2.93)
**Referenced from the whole working population**			
Liver and intrahepatic bile ducts neoplasms	1.15 (1.04–1.27)	1.14 (0.99–1.30)	1.61 (1.50–1.72)
Other digestive organs neoplasms	1.28 (1.04–1.57)	1.37 (1.03–1.79)	1.79 (1.53–2.08)
Trachea, bronchus and lung neoplasms	1.28 (1.15–1.43)	1.25 (1.07–1.44)	1.80 (1.66–1.95)
Bladder neoplasms	1.26 (1.03–1.52)	1.29 (1.01–1.65)	1.72 (1.49–1.98)

First 1-year fixed RTWs: RTWs who worked in the road transport industry during the first year (2006) of the study.

First 3-year fixed RTWs: RTWs who worked in the road transport industry for the first 3 years (2006–2008) of the study.

More than 1 year as RTWs indicated those who worked 1 year or more in the road transport industry during the study (2006–2015).

*RTW* road transportation worker.

Comparison of the health status using the national health examination between RTWs and the whole working population is found in Supplementary Table 1. Most health measures were statistically similar in RTWs and the whole working population group. When examining HDL-cholesterol level and AST level, the RTW group was healthier than the whole working population, which differed from our expectation (Supplementary Table 1). Only regular exercise levels were significantly lower in RTWs than in the whole working population.

## Discussion

Our national representative cohort analysis highlighted that RTWs have a high risk of cancer, including cancer of the liver and intrahepatic bile ducts, other digestive organs, trachea, bronchus, lung, and bladder. Those relationships were not altered after a sensitivity analysis accounting for job exposure change.

Various articles show that professional drivers are exposed to occupational hazards such as shift work, long working hours, biochemicals and toxic materials, social isolation, and lack of decision-making authority^16^. Consequently, these hazardous factors can result in certain physical (cardiovascular disease, gastrointestinal disorders, musculoskeletal problems, and cancer), psychological (depression, anxiety, and post-traumatic stress disorder), and behavioural (substance abuse) outcomes^17–20^.

Engine exhaust is particularly thought to cause lung cancer in RTWs^21^. Working groups convened by the IARC reviewed the scientific literature and classified DEE as ‘carcinogenic to humans’ (Group 1) and gasoline engine exhaust as ‘possibly carcinogenic to humans’ (Group 2B)^22^. A large retrospective study reported that trucking industry workers who had regular exposure to vehicle exhaust from diesel and other types of vehicles on highways, city streets, and loading docks have a 15–40% increased risk of lung cancer as years of work increased^23^. When this study was extended with an exposure assessment on the basis of elemental carbon, lung cancer mortality in trucking industry workers was found to be increased in association with cumulative exposure to elemental carbon after adjusting for employment duration^24^. Other occupational cohort and case–control studies including various occupations involving exposure to diesel engine exhaust, also supported the findings of these cohort studies on truck industry workers^25^. Furthermore, the risk of lung cancer was positively associated with exposure to nitrogen dioxide, nitrogen oxide, sulphur dioxide, and fine particulate matter. A meta-analysis showed that occupational exposure to air pollution among professional drivers significantly increased the incidence (meta‐odds ratio [OR] 1.27, 95% CI 1.19–1.36) and mortality (meta‐OR 1.14, 95% CI 1.04–1.26) of lung cancer^26^.

An increased risk of bladder cancer among RTWs has also been reported in many case–control studies, although such risks were not definite in cohort studies. A meta-analysis of 3 cohort studies and 27 case–control studies during 1977–2008 indicated an elevated bladder cancer risk among RTWs and railroad workers^27^. The RTWs had a higher risk for sedentary lifestyle at the workplace due to their working conditions. Previous studies have reported that sedentary lifestyles are closely linked to various cancers, including bladder cancer^28,29^. Sedentary behaviours might be related to both increased levels of pro-inflammatory factors and decreased levels of anti-inflammatory factors^30^. Furthermore, physical activity may reduce systemic inflammation by reducing adipose-related inflammatory cytokines^31^. RTWs were exposed to chronic sedentary working conditions, which might be a risk factor for cancer.

Liver and intrahepatic bile duct malignancies are a leading cause of death worldwide, and hepatocellular carcinoma (HCC) is a representative malignancy among liver cancers^32^. The most common causes of liver cancer are lifestyle factors such as alcohol drinking (even moderate) and hepatitis B and C viral infections^32–34^. Chronic inflammation and oxidative stress are major intermediate aetiologies bridging environmental risk factors and carcinogenesis^33^. Hence, antioxidants may lower the liver cancer incidence, and an animal study showed that dietary antioxidants reduced the risk of HCC in a viral hepatitis animal model^33,35^. A recent review article suggested carcinogenic chemicals as a possible aetiology for liver malignancy, including acrylamide perfluorooctanoic acid, polychlorinated biphenyls, benzo(a)pyrene, perfluorinated chemicals, and vinyl chloride monomers^36^. These chemicals can cause cytotoxicity and DNA damage, which may lead to liver malignancy.

The results of our study show an increased risk of liver cancer among RTWs. Some studies have supported that relationship, indicating an aetiology of liver cancer due to exposure to fine particles, air pollution, and DEE. A case–control study of 314 Chinese HCC patients showed that patients exposed to indoor air pollution had four times higher odds of developing HCC. The OR with 95% CI was 2.46 (1.47–4.14) after adjustment for hepatitis B viral infection, liver disease, exposure history of pesticide, and other basic demographic characteristics^37^. A cohort study with more than 15 years of follow-up, including 464 HCC cases in Taiwan, assessed exposure of fine particulate matter (PM2.5) using regional monitoring data and patients’ residential addresses^38^. The authors reported that PM2.5 exposure was related to incidence of HCC via an indirect effect of elevated serum alanine transaminase and suggested that PM2.5 causes systemic inflammation, which elevates serum alanine transaminase levels and can cause HCC. The main target organ of fine particles, including DEE, is the respiratory tract, but inhaled fine particles can translocate and enter the systemic circulation^39^. Exposure to DEE via oral consumption increased DNA adducts, DNA damage, and apoptosis in liver cells in an animal study^11^. A molecular epidemiological case–control study collected liver tissue from HCC patients and analysed DNA adduction by PAHs, which are a component of DEE and well-known carcinogens of the lung and bladder^40^. The OR (95% CI) of HCC was 3.9 (1.0–14.9) in the third tertile for DNA-PAH adduction after adjusting for age, sex, and HBsAg. Interestingly, the odds of HCC were elevated 20 times by DNA-PAH adduction in HBsAg-positive patients. Systemic circulation and DNA adduction of fine particles and DEE can explain the increased risk of liver cancer in RTWs in the present study.

Our large sample size study showed a relationship between exposure in RTWs and various cancer risks. However, it had several limitations. The lack of quantitative exposure assessment is our main limitation. Various occupational hazardous factors such as chemicals, dust, and sedentary duration at work can be potential risks or mediating factors for cancer, and RTWs are generally exposed to these hazardous factors. Furthermore, well-structured job-exposure matrices for specific work^41^ have not been established for RTWs in Korea. The dose–response relationship could not be analysed in this study due to a lack of information regarding the exact exposure levels of various occupational hazardous factors. Further studies with a more comprehensive exposure assessment are needed. Furthermore, we did not employ a job-exposure matrix such as the NOCCA-JEMs^15^, which is the most fundamental method for exposure assessment. In addition, lifetime and cumulative exposure were not included in this study. Although all three scenario approaches of cohort definition show almost same statistical results, more comprehensive and quantitative exposure assessments are needed to clarify the association between RTWs and the risk of various cancer. Additionally, cancer incidents are defined when there are hospital admission histories. Hence, if patients with cancer visit only outpatient clinics or die without admission, they would not be recorded and there would be an underestimation of incident cases. Those underestimations have no direction with regards to the exposed group or the non-exposed group; hence, non-differential misclassification may have occurred. Consequently, the risk of cancers might instead be a forward to null relationship.

In conclusion, RTWs have a high risk of cancer, including cancer of the liver and intrahepatic bile ducts, other digestive organs, trachea, bronchus, lung, and bladder. Particularly, our results suggested that the IARC should again review the relationship between RTWs and risk of bladder cancer. Cancer researchers should address various risks of cancer in RTWs. We believe our results can assist to construct preventive strategies for various cancers in RTWs.

## Methods

### Ethical consideration

Data of this study were anonymised prior to release to the authors from the National Health Insurance Service (NHIS). The Institute Review Board (IRB) of the Yonsei University Health System, Seoul, Korea, approved this study design (IRB number: Y-2017-0100). This data is secondary data from the Korean NHIS database; therefore, informed consents was not needed.

### Data collection

We used data from the Korean NHIS database from 2002 to 2015. The NHIS provides mandatory public health insurance and offers coverage of medical care services, including national health insurance, medical aid, and long-term care insurance, to all Korean citizens^42^. The entire population residing within the territory of Korea is covered by the National Health Insurance of Korea^43^, and all citizens of Korea are expected to join the National Health Security System^44^.

All hospital facility visiting information from the Korean NHIS database were categorised under the standardised protocol of the Korea Classification of Diseases and Causes of Death 4th edition, which corresponds to the International Classification of Diseases, 10th revision (ICD-10). All diagnoses were described based on the ICD-10 codes. The NHIS database included qualification and claims medical service data. The qualification data in the NHIS database included age, sex, region, income, type of insurance, identification number, and family information. Medical service data had records of all covered inpatient and outpatient visits, procedures, and prescriptions. The NHIS provides, free of charge, annual or biennial health screening examinations that include the assessment of chronic disorders, mental health, and lifestyle. We used this health screening examination data to compare lifestyle differences between RTWs and the general population.

### Study participants

The NHIS database included 50,908,646 patients in 2011, 51,169,141 in 2012, 51,448,491 in 2013, 51,757,146 in 2014, and 52,034,424 in 2015, which covers approximately 98% of the people living in the territory of Korea^45^. There are two main services in the NHIS in Korea, 97% makes up the medical care sector and 3% of medical aid sector^46^. Medical care includes four types of insurances: employee subscriber, employee dependent, district subscriber, and district dependent. To select the RTWs, first we selected male participants aged between 25 and 69 years with NHIS eligibility as employee subscribers in 2006. Subsequently, we excluded participants who had claims for any cancer from 1 January 2002 to 31 December 2005 to ensure first admission history as the incident case. To select the RTW group, we first defined the transportation working group as those working in section H ‘transportation and storage’ from the most recent Korean national standardised industrial classification, which was developed by the Korea National Statistical Office following the 4th revision of the International Standard Industrial Classification of All Economic Activities in 2008^47^. This section includes the provision of passenger or freight transport by rail, pipeline, road, water, or air and associated activities. RTWs were defined as only those among the workers involved in road transportation. This group includes all land-based transport workers such as bus drivers, taxi drivers, shuttle drivers, others who drive rented private cars, and all freight transport operators on the road. Then we defined the RTWs using three deferent definitions based on job duration during follow-up periods due to job changing, detailed information was in ‘Definition of job exposure’ section. To demonstrate the risk of cancers in RTWs compared with other populations, we used two reference populations: a healthy working population and a general working population. Government employees were selected as reference for a healthy working population, including public officers or private school staff members who were part of the NHIS and paid by governmental employee pension services. The working population eligible to be employee subscribers were selected as reference for the general working population. From 2006 to 2015, 594,629 person-years of observation were applied for the RTWs (target working population), 3,268,312 person-years for government employees (reference for healthy working population), and 62,760,615 person-years for the entire working population (reference for general working population).

### Definition of job exposure

A major obstacle of occupation-based studies is that workers frequently change industries or exit and re-enter worksites. Previous studies that focused on working populations used combined methods of assessing longest-occupational class cross-classified by industry sector to evaluate factors associated with job exposure^48–50^. We applied three different assessments of job exposure using modified methods: (1) the first 1-year fixed RTWs, in which RTWs were defined as road transport industry workers in the first year (2006) of the study; (2) the first 3 years fixed RTWs, in which RTWs were defined as workers who continuously worked in the road transport industry for the first 3 years (2006–2008) of the study; and (3) the dynamic cohort, made up of those who changed jobs every year. RTWs were defined as workers in the RTW industry during the 2006 to 2014 period, and the person year of cohort were estimate the ‘enrol year’ and cancer incidence, census, or last follow-up of 2015. The reference group was defined as same logic for each scenario cohort.

### Cancers

The NHIS claims for inpatient and outpatient visits, procedures, and prescriptions were coded using the ICD-10, which was adopted in Korea in 1995, and renamed as the Korean Drug and Anatomical Therapeutic Chemical Codes^51^. Cancers were defined those with claims information indicating C00–C97 malignant neoplasm ICD-10 codes. During the follow-up period, only the first diagnosed cancer for each individual was included. Ninety-eight cancers were categorised into 27 categories based on the Korean Standard Classification of Diseases from the ICD-10 according to human organ system^52,53^ as follows: malignant neoplasm of the lip, oral cavity and pharynx (C00–C14), oesophagus (C15), stomach (C16), colon (C18), rectosigmoid junction, rectum, anus, and anal canal (C19–C21), liver and intrahepatic bile ducts (C22), pancreas (C25), other digestive organs (C17, C23, C24, C26), larynx (C32), trachea, bronchus, and lung (C33, C34), other respiratory and intrathoracic organs (C30, C31, C37–C39), bone and articular cartilage (C40, C41), malignant melanoma of the skin (C43), other skin (C44), mesothelial and soft tissue (C45–C49), breast (C50), prostate (C61), other male genital organs (C60, C62, C63), bladder (C67), other urinary tract (C64–C66, C68), brain (C71), other parts of the central nervous system (C70, 72), Hodgkin diseases (C81), non-Hodgkin lymphoma (C82–C86), leukaemia (C91–C95), other lymphoid, haematopoietic, and related tissue (C88–C90, C96), and other, ill-defined, secondary, unspecified, and multiple sites (C73–C80, C97). We excluded female genital organ cancers (C51–58) and malignant neoplasms of the eye and adnexa (C69), which were not present in any participants.

### Health status

To consider health status by occupation, we used the oldest national health screening examination data that could demonstrate the health status of workers before cancer diagnosis during the study period. A total of 549,222 workers and 5,380 RTWs participated in national health screening examinations. These data included information regarding health behaviour (smoking, alcohol drinking, and exercise level) and anthropometric data (body mass index (BMI)), fasting glucose level, lipid profile, liver function, and blood pressure (hypertension).

### Statistical analysis

The crude incidence, SIR, and 95% CI of all cancer risks among the RTW were compared to those of the whole working population after adjusting for age (5-year range) using a direct standardised method by the following year. To calculate direct standardisation, the age-specific cancer incidence was calculated for each 5-year age group among the RTW. These values were then multiplied by age-specific cancer incidence rates and the number of person-years in each age group of the whole working population (the reference group), which made the age-specific expected incidence case. The addition of all age-specific expected cases indicated the total incidence. The ratio of the observed and expected numbers of cases was the direct age-SIR. The whole working population and government employees were used as reference groups, separately for each analysis. If the SIR was more than 1 and the lower limit of the 95% CI was also more than 1, the cancer risk was considered significantly higher in the RTW group than in the reference groups. A mid-P test was used for calculating 95% CIs. For the sensitive analysis, we used three different definitions of the cohort to estimate SIRs and 95% CIs for cancer risk with statistical significance above analysis.

To demonstrate effects of confounding variables, we compared BMI, fasting glucose level, lipid profile, liver function, hypertension, smoking, alcohol drinking, and exercise between RTWs and the whole working population using Student’s t-test or chi-squared test. All analyses were conducted using SAS, version 9.4 (SAS Institute, Cary, NC, USA).

### Ethical consideration

Data of this study were anonymised prior to release to the authors from the NHIS. The Institute Review Board (IRB) of the Yonsei University Health System, Korea approved this study design (IRB number: Y-2017-0100). This data is secondary data from NHIS, hence informed consent was not needed in current study.

## Supplementary information

Supplementary Information.

## Data Availability

Data cannot be available in public.
