# Supplementary material for: Cancer risk in road transportation workers: a national representative cohort study with 600,000 person-years of follow-up
Source: Sci Rep. 2020 Jul 9;10:11331. doi: 10.1038/s41598-020-68242-5 (PMC7347601; doi:10.1038/s41598-020-68242-5)
Supplement: Supplementary file 1 — Supplementary Information. [file 41598_2020_68242_MOESM1_ESM.docx]

**Title**

Cancer risk in road transportation workers: A national representative cohort study with 600,000 person-years of follow-up

**Authors**

Wanhyung Lee^1^, Mo-Yeol Kang^2^, Jihyun Kim^3,4^, Sung-Shil Lim^3,4^ and *Jin-Ha Yoon^4,5^

**Affiliations**

^1^Department of Occupational and Environmental Medicine, Gil Medical Center, Gachon University College of Medicine, Incheon, Republic of Korea

^2^Department of Occupational and Environmental Medicine, Seoul St. Mary’s Hospital, College of Medicine, The Catholic University of Korea, Seoul, Republic of Korea

^3^Graduate School of Public Health, Yonsei University, Seoul, Republic of Korea.

^4^The Institute for Occupational Health, Yonsei University College of Medicine, Seoul, Republic of Korea

^5^Department of Preventive Medicine, Yonsei University College of Medicine, Seoul, Republic of Korea

***Correspondence to this address:**

Jin-Ha Yoon M.D., Ph.D.

The Institute for Occupational Health, Department of Preventive Medicine,

Yonsei University College of Medicine

50, Yonsei-ro, Seodaemun-gu, Seoul, Korea, [03722]

Tel: +82-2-2228-1579; Fax: +82-2-392-8622

E-mail: [flyinyou@yuhs.ac](mailto:flyinyou@yuhs.ac)

Supplementary Table S1

| **Supplementary Table S1.** Characteristics of workers included in the subgroup analysis based on the national health examination | | | |
| --- | --- | --- | --- |
|  | Road transport workers  (n=5,380) | Whole working population  (n=549,222) | p-value |
| Body mass index (kg/m^2^) | 23.94 ± 0.04 | 24.01 ± 0.01 | 0.0731 |
| Fasting glucose level (mg/dL) | 102.60 ± 0.39 | 102.70 ± 0.04 | 0.7858 |
| Lipid profile (mg/dL) |  |  |  |
| Total cholesterol | 197.50 ± 0.52 | 198.20 ± 0.05 | 0.2085 |
| Triglyceride | 135.90 ± 1.48 | 135.60 ± 0.15 | 0.8246 |
| LDL-cholesterol | 116.00 ± 0.68 | 115.50 ± 0.07 | 0.4154 |
| HDL-cholesterol | 56.45 ± 0.81 | 53.60 ± 0.04 | 0.0004 |
| Liver enzyme (U/L) |  |  |  |
| AST | 26.90 ± 0.19 | 27.40 ± 0.02 | 0.0105 |
| ALT | 24.37 ± 0.19 | 24.60 ± 0.03 | 0.3328 |
| ɤ-GTP | 26.71 ± 0.72 | 37.80 ± 0.08 | 0.1336 |
| Hypertension |  |  | 0.0515 |
| Yes | 1,533 (28.49) | 163,192 (29.71) |  |
| No | 3,847 (71.51) | 386,030 (70.29) |  |
| Smoking |  |  | 0.1422 |
| Never | 3,603 (69.13) | 377,503 (70.73) |  |
| Past | 787 (15.10) | 71,713 (13.44) |  |
| Current | 822 (15.77) | 84,522 (15.84) |  |
| Alcohol drinking |  |  | 0.6513 |
| None to social | 4,173 (78.43) | 424,154 (78.17) |  |
| Severe | 1,148 (21.57) | 118,465 (21.83) |  |
| Exercise |  |  | 0.0191 |
| Irregular | 1,476 (58.55) | 148,211 (56.22) |  |
| Regular | 1,045 (41.45) | 115,408 (43.78) |  |
